# „Mit Bluetooth ein Signal der Solidarität senden“? – Eine medizinethische Analyse der öffentlichen Debatte über die Corona-Warn-App

**DOI:** 10.1007/s00481-023-00751-z

**Published:** 2023-02-28

**Authors:** Niklas Ellerich-Groppe

**Affiliations:** Abteilung Ethik in der Medizin, Carl von Ossietzky Universität Oldenburg, Ammerländer Heerstraße 114–118, 26129 Oldenburg, Deutschland

**Keywords:** Solidarität, Corona-Warn-App, Digitale Gesundheitsversorgung, Contact-Tracing, Öffentlicher Diskurs, Public Health, Solidarity, Corona-Warn-App, Digital healthcare, Contact tracing, Public discourse, Public health

## Abstract

In der öffentlichen Debatte über die Corona-Warn-App kann der Solidaritätsbegriff als wichtiger, aber inhaltlich umstrittener normativer Bezugspunkt gelten. So stehen hier unterschiedliche Solidaritätsrekurse mit heterogenen Voraussetzungen, normativen Implikationen und praktischen Konsequenzen nebeneinander, die einer medizinethischen Untersuchung bedürfen. Vor diesem Hintergrund ist es Ziel des Beitrags, *erstens* die Bandbreite der Verwendungsweisen des Solidaritätsbegriffs in der öffentlichen Debatte zur Corona-Warn-App anschaulich zu machen sowie *zweitens* die Voraussetzungen und normativen Implikationen dieser Verwendungsweisen herauszuarbeiten und einer ethischen Bewertung zu unterziehen.

Dazu stelle ich nach einer kurzen Einführung in die Corona-Warn-App und einer Vergegenwärtigung der Grundzüge des Solidaritätskonzepts vier Beispiele aus der öffentlichen Debatte zur Corona-Warn-App dar, die mit Blick auf die zugrundeliegende Identifikation, die Solidaritätsgruppe, den solidarischen Beitrag sowie das normative Ziel erhebliche Unterschiede aufweisen. Sie unterstreichen die Notwendigkeit weiterführender Maßstäbe, um ihre Legitimität zu bewerten. Dazu greife ich auf vier normative Kriterien einer kontextsensitiven, moralisch gehaltvollen Solidaritätskonzeption zurück (Solidaritätsoffenheit, gestaltbare Inklusivität, Angemessenheit des solidarischen Beitrags, normative Abhängigkeit) und evaluiere auf dieser Grundlage die vorgestellten Solidaritätsrekurse ethisch.

Für alle dargestellten Solidaritätsrekurse lassen sich in der Folge kritische Rückfragen formulieren. Dabei werden einerseits die Potenziale und Limitationen von Solidaritätsrekursen in öffentlichen Debatten deutlich. Andererseits werden Schlussfolgerungen möglich, wann eine Tracing-App tatsächlich als solidarische Technologie zur Pandemiebekämpfung verstanden werden kann.

## Abstract (en)

**Definition of the problem:**

In the public debate in Germany on the Corona-Warn-App, the concept of solidarity is a prominent, but contested normative reference point. Thus, different uses of the concept with heterogeneous assumptions, normative implications and practical consequences stand next to each other and require medical ethical investigation. Against this backdrop, this contribution aims firstly to illustrate the spectrum of understandings of the concept of solidarity in the public debate on the Corona-Warn-App. Secondly, it elaborates the preconditions and normative implications of these uses and evaluates them from an ethical perspective.

**Arguments:**

Starting with an introduction of the Corona-Warn-App and a general definition of the concept of solidarity, I present four examples for different uses of the concept of solidarity from the public discourse on the Corona-Warn-App that vary regarding the underlying identification, the group of solidarity, the solidarity contribution and the normative goal. They highlight the need for further ethical standards in order to assess their legitimacy. Hence, I use four normative criteria of a context-sensitive, morally substantial conception of solidarity (openness, malleable inclusivity, adequacy of the contribution, normative dependence) to ethically evaluate the solidarity recourses presented.

**Conclusion:**

Critical remarks can be formulated for all presented notions of solidarity. On the one hand, the potentials and limitations of solidarity recourses in public debates become apparent. On the other, criteria can be derived for a solidarity-promoting use of the Corona-Warn-App.

## Einleitung: Solidarität und die Corona-Warn-App – eine medizinethisch relevante Debatte

„Solidarisch: Jeder kann zum besseren Schutz aller beitragen.“ So bewarb die Bundesregierung die Corona-Warn-App kurz nach deren Einführung in den Sozialen Medien (Bundesregierung 2020a). Schon als die App am 16. Juni 2020 nach langer, kontroverser Diskussion vorgestellt worden war, hatte sich Kanzleramtsminister Braun in ähnlicher Richtung geäußert: „Sie herunterzuladen ist ein kleiner Schritt für jeden von uns, aber ein großer Schritt für die Pandemiebekämpfung“ (Bundesregierung 2020b). Auch weitere Politiker*innen wie Innenminister Seehofer stellten die App als neues Instrument der Infektionsnachverfolgung in die lange Reihe der als solidarisch klassifizierten Maßnahmen zur Pandemiebekämpfung, wie dem Maskentragen und besonderer Hygiene (Redaktionsnetzwerk Deutschland 2020).

In der öffentlichen Debatte zur Corona-Warn-App ließ sich damit die Fortführung der impliziten und expliziten Solidaritätsrhetorik beobachten, die den öffentlichen Diskurs in der Pandemie von Beginn an prägte (z. B. Bundeskanzlerin 2020). Bereits zuvor war in Politik, Medien und Zivilgesellschaft regelmäßig dazu aufgerufen worden, durch Vorsicht und Verzicht einen solidarischen Beitrag zu leisten, um gemeinsam das Virus zu bekämpfen und v. a. alte und vulnerable Personen zu schützen (Ellerich-Groppe et al. 2021). Zugleich wurde im weiteren Pandemieverlauf zunehmend unklarer, wer denn nun mit wem auf welcher Grundlage solidarisch sein solle; der Nutzen der Solidarität wurde immer mehr mit ihren Kosten abgewogen. Mitunter fanden sich gar Diagnosen einer Erosion von Solidarität und Warnungen vor einer Diskriminierung der Schwächeren (z. B. August 2020).

Gleiches gilt für die öffentliche Debatte um die Corona-Warn-App, in der der Solidaritätsbegriff bis heute einen wichtigen Bezugspunkt darstellt. Auch hier erfolgten die Bezüge aus sehr unterschiedlichen Positionen, mit heterogenen Interessen und mannigfaltigen praktischen Konsequenzen. So war eine Vielzahl von Appellen zur App-Nutzung zu beobachten, die sich z. B. stark darin unterschieden, wer aus Solidarität mit wem die App nutzen soll. Auch die Frage, wie freiwillig oder verpflichtend eine (solidarische) App-Nutzung sein soll, wurde sehr unterschiedlich beantwortet. Zugleich standen diesen Solidaritätsappellen Warnungen vor einer Entsolidarisierung sowie vor einer Diskriminierung und Stigmatisierung derjenigen gegenüber, die die App nicht nutzen (wollen) oder potenziell erkrankt sind (etwa Dachwitz et al. 2020; Littger 2020). Abermals war Solidarität in aller Munde, ohne dass ein einheitliches Verständnis davon vorherrschte.

Medizinethisch ist diese Debatte in mindestens zweifacher Hinsicht relevant. Sie ist medizin*ethisch* relevant, da in ihr Situationsbeschreibungen, moralphilosophische Überzeugungen und politische Konsequenzen artikuliert und in Zusammenhang gebracht werden. Eine ethische Untersuchung kann hier helfen, „diejenigen Werte und Normen explizit [zu] formulier[en] und diskutier[en], die […] häufig nur implizit und durch die Vermittlung komplexer und dichter Begriffe präsent und wirksam sind“ (Müller-Salo 2020, S. 22). Ethik kommt somit zunächst die Aufgabe des Explizitmachens gesellschaftlich relevanter Werte und Normen zu, die im Rahmen eines bestimmten Diskurses zutage treten. Sodann kann sie die Verwendung dieser Begriffe auf Kohärenz und Legitimität sowie die darin artikulierten Normen und Werte auf ihre Berechtigung prüfen und so originär ethische Anliegen verfolgen. Im Zusammenhang mit der Corona-Warn-App findet sich eine heterogene Verwendung dichter Begriffe, die explizit gemacht und ethisch bewertet werden muss, wie gesehen v. a. mit Blick auf den Solidaritätsbegriff.

Die Debatte ist zudem *medizin*ethisch relevant, da sie wesentlich das gesellschaftliche und politische Handeln in einem für Medizin und Gesundheitsversorgung relevanten Feld abbildet und in der Folge die öffentliche Wahrnehmung – und einen Schritt weitergedacht: das entsprechende Handeln – beeinflusst (Henderson und Hilton 2018). Gerade in einer Pandemie, in der ständig weitreichende individuelle und politische Entscheidungen für die Gesundheitsversorgung getroffen werden, ist eine kritische Analyse der öffentlichen Debatte aus medizinethischer Perspektive daher wichtig. So wie insgesamt mediale Berichterstattungen einen erheblichen Einfluss auf die Bereitschaft haben, neue Public-Health-Maßnahmen zu akzeptieren (Hilton et al. 2014), hängt die Effektivität der Corona-Warn-App davon ab, wie viele Personen sich – auch angesichts der öffentlichen Debatte – von der Nutzung überzeugen lassen. Medizinethische Diskurse haben so konkrete Relevanz und Auswirkungen für Medizin und Gesundheitsversorgung.

In diesem Sinne möchte der vorliegende Beitrag die Verwendung des Solidaritätsbegriffs in der öffentlichen Debatte zur Corona-Warn-App medizinethisch untersuchen. In diesem Zuge zielt er *erstens* darauf, die unterschiedliche Aneignung und die Bandbreite der Verwendungsweisen des Solidaritätsbegriffs durch verschiedene Akteur*innen in verschiedenen Bereichen der öffentlichen Debatte zur Corona-Warn-App explizit und anschaulich zu machen. *Zweitens* sollen die Voraussetzungen und normativen Implikationen dieser Verwendungsweisen herausgearbeitet und einer ethischen Bewertung unterzogen werden, um daraus Schlüsse für die Verwendung von Solidaritätsrekursen in öffentlichen Debatten insgesamt ziehen zu können. Dazu gehe ich folgendermaßen vor: Nach einigen Hinweisen zur Corona-Warn-App und einer kurzen Vergegenwärtigung der Grundzüge des Solidaritätskonzepts werden sodann vier Beispiele aus der öffentlichen Debatte diskutiert. Sie unterstreichen die Notwendigkeit weiterführender Maßstäbe, um ihre Legitimität zu bewerten. Unter Rückgriff auf vier normative Kriterien einer kontextsensitiven, moralisch gehaltvollen Solidaritätskonzeption ist es in einem letzten Schritt möglich, die vorgestellten Solidaritätsrekurse ethisch zu evaluieren und kritisch hinsichtlich ihrer Potenziale und Limitationen einzuordnen. Zudem werden erste Schlussfolgerungen möglich, wann eine Tracing-App tatsächlich als solidaritätsfördernde Technologie zur Pandemiebekämpfung verstanden werden kann.

## Digitale Technologien in der Pandemiebekämpfung – die Corona-Warn-App

„Digitale Anwendungen können die bisherigen Maßnahmen zur Eindämmung von COVID-19 sinnvoll ergänzen“ (Robert-Koch-Institut 2020). Mit dieser Äußerung unterstrich der Leiter des Robert-Koch-Instituts, Lothar Wieler, bereits im Frühjahr 2020 die Bedeutung digitaler Technologien in der Pandemiebekämpfung. So können digitale Technologien auf vielfältige Weise die weiteren Public-Health-Maßnahmen zur Pandemiebekämpfung unterstützen, z. B. in der raschen Identifizierung von Infektionsfällen und -clustern, der Kommunikation mit der Bevölkerung oder der Bewertung der Wirksamkeit von Maßnahmen (Budd et al. 2020).

Unter den verschiedenen digitalen Möglichkeiten der Pandemiebekämpfung mit ihren je eigenen ethischen Herausforderungen (zum Überblick: Gasser et al. 2020) kommt der digitalen Kontaktverfolgung eine besondere Bedeutung zu, kann doch eine effektive und schnelle Kontaktnachverfolgung als „Eckpfeiler“ der Public-Health-Maßnahmen gelten (Parker et al. 2020, S. 427). Dabei können insbesondere sogenannte Contact-Tracing-Apps ein wichtiger Baustein sein (Jahnel et al. 2020; Ferretti et al. 2020). Entsprechend früh wurde auch in Deutschland die Entwicklung einer solchen App vorangetrieben, die schließlich im Juni 2020 in der Einführung der Corona-Warn-App mündete. Als Ergänzung der analogen Kontaktverfolgung soll sie Menschen mit einem Risikokontakt warnen und dadurch helfen, Infektionsketten frühzeitig zu unterbrechen. Dazu macht sich die App die Bluetooth-Technologie zunutze (vgl. hierzu Bundesregierung 2020c): Bei eingeschalteter Bluetooth-Verbindung registriert die App Smartphones in der Nähe und tauscht einen pseudonymisierten digitalen Schlüssel aus. Stellt ein*e App-Nutzer*in nun eine eigene Coronavirus-Infektion fest, kann die Person ihren positiven Corona-Test in der App hinterlegen und ihre eigenen Zufallsbegegnungen allen anderen App-Nutzer*innen zur Verfügung stellen. Die App-Nutzer*innen, die in den vergangenen Tagen über einen entsprechend langen Zeitraum mit kurzem Abstand mit der infizierten Person in Kontakt waren, werden sodann über ihre Risikobegegnung informiert, können sich selbst isolieren und sich testen lassen. Der dezentrale Aufbau der Datenbasis bei der Corona-Warn-App – nur die pseudonymisierten Schlüssel werden lokal gespeichert und nach expliziter Freigabe der positiv getesteten Person ausgetauscht, es ist keine persönliche Identifizierung möglich – informiert damit nur über Risikobegegnungen. Ein Contact Tracing im engeren Sinne, bei dem die Risikobegegnungen zentral gespeichert werden und tatsächlich durch Dritte identifiziert werden können, ist hingegen nur bei einer zentral aufgebauten Datenbank möglich (Ranisch et al. 2021).

Die Entwicklung und Einführung der App wurden von breiten gesellschaftlichen Debatten begleitet, in denen neben technischen Aspekten prominent die ethischen Herausforderungen einer Tracing-App diskutiert wurden (zum Überblick: Simon und Rieder 2021). Diese öffentlichen Diskurse zu Contact-Tracing-Apps in verschiedenen Ländern sind mittlerweile vielfach Gegenstand wissenschaftlicher Analyse geworden (etwa Rowe et al. 2020; Samuel und Lucivero 2022). Für den deutschsprachigen Raum (Deutschland, Österreich und die Schweiz) lassen sich dabei zu Beginn der Pandemie im Zeitraum von Mitte März bis Anfang Mai 2020 vor allem drei wesentliche Framings von Contact-Tracing-Apps herausarbeiten: Neben einer kritischen Perspektive auf die Implikationen einer solchen App für die Privatsphäre und den Datenschutz identifizieren die Autor*innen einer ersten Erhebung *zweitens* den kontrastierenden Vergleich mit den digitalen Überwachungstechnologien in asiatischen Ländern wie China sowie *drittens* eine länderspezifische Schwerpunktsetzung auf bestimmte politische und wissenschaftliche Akteur*innen (Zimmermann et al. 2021). Dies deckt sich mit den Ergebnissen einer weiteren Analyse, in der sich vor allem sechs Themen im medialen Diskurs als relevant erweisen: Mit Blick auf die App-Entwicklung werden die Rolle großer IT-Unternehmen, die wissenschaftliche Genauigkeit sowie Fragen der Datenverarbeitung und -verwaltung zum Thema; hinsichtlich der App-Nutzung setzt sich die Berichterstattung vor allem mit Fragen der Freiwilligkeit, mit der Funktionalität bzw. Effektivität der App und mit ihrer Rolle in der Pandemiebekämpfung auseinander (Amann et al. 2021).

Wie im medialen Diskurs können auch im fachwissenschaftlichen, ethischen Diskurs Fragen der Privatsphäre und des Datenschutzes als ein Debattenschwerpunkt gelten, der mitunter weitere ethische Aspekte wie die Rolle privater Technologiekonzerne in der App-Entwicklung zu überlappen drohte (Sharon 2020; van Basshuysen und White 2021). Dies geschah vor allem entlang der Unterscheidung zwischen dezentralen und zentralen Ansätzen für eine Tracing-App. Einerseits scheint der dezentrale Ansatz als vermeintlich „privacy preserving by design“ (White und van Basshuysen 2021a) deutlich geeigneter, Fragen der Privatsphäre und des Datenschutzes zu begegnen, die immerhin als entscheidende Elemente der in Europa entwickelten Tracing-Apps gelten können (Blassime et al. 2021, S. 8). Andererseits gilt, dass auch diese Systeme Datenschutzverletzungen nicht vollkommen ausschließen können und ebenso zentrale Systeme sehr datenschutzsicher gestaltet werden können (White und van Basshuysen 2021a, b; van Basshuysen und White 2021). Gleichzeitig erlauben zentrale Ansätze tendenziell eine schnellere, verlässlichere und damit wirksamere Kontaktverfolgung (van Basshuysen und White 2021), sodass sich angesichts dieser erheblich gesteigerten Effektivität und unerheblich unterschiedlichen Datenschutzrisiken auch gute Gründe für die Entscheidung für einen zentralen Ansatz finden lassen (siehe etwa White und van Basshuysen 2021b).

Für beide Ansätze – für den dezentralen noch mehr als für den zentralen (Hernández-Orallo et al. 2020) – gilt jedoch, dass die Anzahl der Nutzer*innen der App entscheidend ist für deren Effektivität. Ein weiterer Schwerpunkt der ethischen Debatte lag deswegen auf der Frage, ob die App-Nutzung freiwillig oder verpflichtend sein solle und inwiefern sie durch Anreize gefördert werden solle. Hier ließ sich eine enorme Bandbreite an Vorschlägen beobachten, die von einem Insistieren auf die absolute Freiwilligkeit der App über eine standardmäßige Aktivierung der App mit Opt-Out-Möglichkeit (etwa Hernández-Orallo et al. 2020) sowie (monetäre) Anreize bis hin zu Argumentationen und Rechtfertigungen für eine verpflichtende oder gar erzwungene App-Nutzung (z. B. Basu 2021) reichten. Eine verpflichtende Nutzung wird dabei tendenziell als einer liberalen Demokratie widersprechend eingeordnet (etwa Lanzing 2021, S. 89). Hingegen werden Anreize für die App begrüßt, solange Fairness-Aspekte berücksichtigt werden und Anreizsysteme nicht bestehende soziale und digitale Ungleichheiten verstärken oder bestimmte Personengruppen unverhältnismäßig stark in ihren Freiheiten beschränken (Loi 2021; Parker et al. 2020). Ein Beispiel für einen solchen Anreiz stellt der Vorschlag eines für die App-Nutzung optimierten, kostenlosen Smartphones dar (Loi 2021). Interessanterweise sehen wieder andere in der App selbst bereits den größten Anreiz, der darin bestehe, das eigene Risiko zu reduzieren, ein schnelleres Ende von Lockdown-Maßnahmen zu erreichen sowie einen Beitrag zum Schutz der Mitmenschen zu leisten (Parker et al. 2020, S. 429). Solidaritätsaufrufe und Verweise auf die gemeinsame Betroffenheit von der Pandemie werden hier als erfolgsversprechendes Mittel verstanden, um eine höhere Nutzer*innenanzahl zu befördern (Parker et al. 2020, S. 429). Angesichts der für eine effektive App notwendigen gesellschaftlichen Akzeptanz und ausreichenden Nutzer*innenanzahl wurde früh auf die Notwendigkeit von Maßnahmen hingewiesen, die eine möglichst umfassende und funktionsgemäße Nutzung befördern (Jahnel et al. 2020, S. 666). Es überrascht daher nicht, dass es etwa den politischen Entscheidungsträger*innen von Anfang an ein Anliegen war, für eine breite Nutzung zu werben. In diesen Appellen kam – wie schon beim Werben für die „analogen“ Schutzmaßnahmen in der Corona-Pandemie – dem Solidaritätsbegriff eine Schlüsselrolle in der öffentlichen Debatte zu. Zugleich wurden in diesem Zusammenhang die verschiedenen ethischen Herausforderungen einer Contact-Tracing-App aufgegriffen und mit dem Solidaritätsbegriff verschränkt, sodass z. B. mal die Möglichkeit einer gleichberechtigten Teilhabe, mal die Freiwilligkeit der App und mal der solidarische Imperativ zur App-Nutzung selbst betont wurde.

## Solidarität: Elemente eines umstrittenen Begriffs

Mit dem Solidaritätsbegriff hat sich ein vieldiskutierter Begriff als normativer Referenzpunkt in der Corona-Pandemie und speziell in der Diskussion um die Corona-Warn-App etabliert. Einmal mehr erweist sich *Solidarität* als Krisenterminus: Auf ihn wird gerade dann rekurriert, wenn die üblichen moralischen und juristischen Verhältnisse einer Gesellschaft fragil oder unvollständig scheinen und die Voraussetzungen von Solidarität scheinbar angegriffen sind (ter Meulen et al. 2001, S. 7). Dieser „moralische[] Ausnahmezustand“ (Derpmann 2013, S. 209), in dem an Solidarität appelliert wird, umfasst somit einen impliziten ethischen Reflexionsappell und mahnt einen moralischen Fortschritt an – Löschke spricht von der „Beseitigung eines identitätsrelevanten moralischen Missstandes“ als Ziel der Solidarität (Löschke 2015, S. 77). Zugleich wird der Begriff sehr uneinheitlich verwendet, sodass die Gefahr besteht, ihn in seiner konkreten Bedeutung zu entleeren.

Wagt man unter Berücksichtigung einschlägiger formaler Definitionen von Solidarität (etwa Bayertz 1998, S. 11; Prainsack und Buyx 2016, S. 82) eine Rekonstruktion des Kerngehalts dieses umstrittenen Begriffs, besteht Solidarität darin, innerhalb oder gegenüber einer Gruppe bzw. gegenüber einer Person einen Beitrag zu leisten, weil man sich aufgrund einer bestimmten Gemeinsamkeit oder relevanten Beziehung mit ihr identifiziert (Ellerich-Groppe 2021). Solidarität ist dabei normativ abhängig; der Begriff entfaltet seine normative Verbindlichkeit erst durch den Bezug auf weitere Prinzipien und Werte, wie Gerechtigkeit oder Freiheit (Löschke 2015; Forst 2021).

Solidarität zeichnet sich also durch einen *Gruppenbezug* aus: Entweder ist man selbst Teil einer Gruppe, deren Mitglieder miteinander solidarisch sind, oder man ist solidarisch mit einer anderen Gruppe bzw. einzelnen Mitgliedern dieser Gruppe (Taylor 2015). Solidarität ist damit partikular: Nicht alle Menschen sind mit allen beliebig solidarisch, mehr noch muss es einen *Identifikationspunkt*, eine spezifische Gemeinsamkeit oder sonstige persönliche Beziehung geben, damit ich mich solidarisch erkläre – der für Solidarität konstitutive Zusammenhang ist subjektiv bedeutsam (Bayertz 1998, S. 12). Diese Identifikation resultiert in der Bereitschaft zur oder in tatsächlich vollzogener Hilfe oder Kooperation; allgemeiner lässt sich von einem *Beitrag* sprechen, der im Rahmen der Solidarität geleistet wird. Dabei ist umstritten, inwiefern solidarisches Handeln eine Verpflichtung ist. So wird Solidarität mal als supererogatorische Handlung verstanden, während andere bei den solidarisch Handelnden zwar eine Verpflichtung zur Solidarität sehen, der aber kein Rechtsanspruch der Rezipient*innen korrespondiert (Wildt 1998, S. 212). Die Frage nach dem Beitrag und dem Verpflichtungsgrad von Solidarität adressiert auf allgemeinerer Ebene das Verhältnis der solidarisch Handelnden untereinander sowie die Frage, was eine solidarische Person legitimerweise von anderen erwarten kann und was sie selbst zu leisten bereit sein muss. Schlussendlich ist die *normative Abhängigkeit* von Solidarität zu betonen. Solidarität erhält ihren normativen Gehalt erst durch die Bezugnahme auf weitere Prinzipien und Werte. Dieser Aspekt ist wesentlich, um die Legitimität von Solidaritätsforderungen zu bewerten, weil er eine moralisch verwerfliche Inanspruchnahme von Solidarität ausschließt (Löschke 2015, S. 71–73).

Diese Elemente von Solidarität bieten einen ersten Anknüpfungspunkt und einen heuristischen Begriffsrahmen, um Solidaritätsverständnisse in öffentlichen Debatten zu identifizieren und zu systematisieren. Die Heterogenität der Verständnisse lässt sich dabei v. a. als Folge einer unterschiedlichen Gewichtung dieser Elemente begreifen (Löschke 2015), die sich auch mit Blick auf die unterschiedlichen Ebenen von Solidarität zeigt: Je nachdem, ob es sich um Solidarität auf der interpersonellen, kollektiven oder rechtlich-politischen Ebene handelt, werden die Elemente anders betont und ins Verhältnis gesetzt (Prainsack und Buyx 2016, S. 83–88).

## Einer für alle, alle für einen? Die Vielfalt der Verwendungsweisen von Solidarität in der öffentlichen Debatte

Entlang des *Gruppenbezugs*, der *Identifikationsgrundlage*, des *Beitrags *sowie der *normativen Abhängigkeit* soll nun anhand von vier Fällen gezeigt werden, wie unterschiedlich der Solidaritätsbegriff in der öffentlichen Debatte um die Corona-Warn-App verwendet wurde. Dabei lege ich ein Verständnis der öffentlichen Debatte zugrunde, das mit dem medialen, dem politischen und dem zivilgesellschaftlichen Diskurs drei Arenen umfasst, die in der öffentlichen Aushandlung während der Corona-Pandemie – auch in anderen Debatten (vgl. etwa Ellerich-Groppe et al. 2021) – relevant wurden und die Allgegenwart impliziter und expliziter Solidaritätsrekurse in unterschiedlichen Diskursbereichen anschaulich machen.

Bei der Auswahl der Fälle geht es nicht um einen repräsentativen Einblick in den öffentlichen Diskurs; es handelt sich um eine theoriegeleitete Auswahl von vier Fällen, die die einzelnen Elemente von Solidarität je unterschiedlich betonen und dadurch auch verschiedene Aspekte der breiteren Debatte um die Corona-Warn-App in den Vordergrund stellen. Um die heterogene Aneignung des Solidaritätsbegriffs im Diskurs durch einzelne Akteur*innen anschaulich zu machen, wird bewusst auf unterschiedliche Quellen zurückgegriffen. In diesem Sinne diskutiere ich als Beispiel aus dem politischen Diskurs zunächst das von Angela Merkel aufgerufene Solidaritätsverständnis der Pandemiebekämpfung als Gemeinschaftsprojekt der Bürger*innen. Aus dem medialen Diskurs stammt sodann das Framing der App-Nutzung als solidarische Bürger*innenpflicht. Hier liegt der Schwerpunkt auf dem Verpflichtungsgrad des solidarischen Beitrags, wohingegen ein weiteres Beispiel aus dem politischen Diskurs mit seinem Fokus auf die freiwillige Identifikation zwischen der Warnung vor Endsolidarisierung und dem Appell an eine umfassendere Solidarität changiert. Steht bei all diesen Rekursen die Solidarität innerhalb einer Gruppe im Vordergrund, betont Diakonie-Präsident Ulrich Lilie als zivilgesellschaftlicher Akteur das Gebot der Solidarität zur App-Nutzung insbesondere vulnerablen Gruppen gegenüber und rückt die Teilhabebarrieren der App sowie auch insgesamt einen anderen Gruppenbezug in den Vordergrund. Auf Grundlage dieser Fälle lassen sich die Vielfalt theoretischer Vorannahmen und normativer Implikationen von Solidaritätsrekursen in der öffentlichen Debatte illustrieren und – nach einer anschließenden ethischen Evaluation – Konsequenzen für eine ethisch gerechtfertigte Verwendung des Solidaritätsbegriffs in öffentlichen Diskursen formulieren.

### Pandemiebekämpfung als Gemeinschaftsprojekt der Bürger*innen

Der erste Solidaritätsrekurs zeigt sich eindrücklich in einer Videobotschaft von Bundeskanzlerin Merkel zur Einführung der Corona-Warn-App. Merkel grenzt gleich zu Beginn durch die Anrede „Liebe Mitbürgerinnen und Mitbürger“ die adressierte Gruppe ein (Merkel 2020, 0:00–0:01 min). Zu der angesprochenen Bürger*innengemeinschaft zählt sie sich auch selbst, was die häufige Nutzung der Pronomen „wir“ und „uns“ im gesamten Video unterstreicht. Sie betont, dass jede*r App-Nutzer*in einen Beitrag dazu leiste, das Virus weiterhin zu kontrollieren (0:15–0:22 min). So wird zugleich das normative Ziel benannt, nämlich die Kontrolle des Virus. Sodann weist sie auf höchste IT-Sicherheit, Datenschutz und Transparenz als Grundzüge der App hin und unterstreicht damit Gesundheitsschutz und Infektionskontrolle als alleinige Ziele der App (0:28–0:36 min). Merkel hebt anschließend den individuellen Vorteil für die einzelnen Nutzer*innen hervor, sich aufgrund einer Warnung durch die App schnell testen lassen und Gewissheit erlangen zu können (1:41 min). So könne man sich und andere schützen (1:41–1:43 min). Zudem betont Merkel die Freiwilligkeit der App, es gebe keine Belohnung für die Benutzung, bei Nicht-Nutzung seien keine Nachteile zu befürchten (2:00–2:10 min). Damit sind wesentliche Grundlagen für eine Identifikation als Solidaritätsgruppe gelegt. Ergänzend führt Merkel weitere „sehr gute Argumente für die App“ (2:13–2:15 min) an: Neben dem eigenen Interesse, von einem Ansteckungsrisiko zu erfahren, betont Merkel den Nutzen, „der für die Gemeinschaft in dieser App steckt“ (2:24–2:26 min). Dieser sei jedoch abhängig von den einzelnen Bürger*innen: „[J]e mehr mitmachen, desto größer ist dieser Nutzen“ (2:26–2:30 min). Im Anschluss rahmt sie die App als konsequente Fortsetzung des als solidarisch klassifizierten Verhaltens in der Pandemiebekämpfung: „Gemeinsam, mit Vernunft und Verantwortungsgefühl haben wir die Ausbreitung des Virus eingedämmt“ (2:30–2:36 min). Es käme nach wie vor auf das Verhalten aller an. Das gemeinsam Erreichte in der Pandemie wird somit zur weiteren Identifikationsgrundlage und die App-Nutzung abermals in eine Reihe mit weiteren – solidarischen – Beiträgen gestellt. Zum Abschluss bittet Merkel „auch diejenigen, die es noch nicht getan haben“ (3:24–3:26 min), die App zu nutzen und dankt dafür im Voraus.

In dieser ersten Verwendungsweise wird somit jedes Mitglied der Bürger*innengemeinschaft (*Gruppenbezug*) aufgerufen, die App als Ergänzung weiterer *Beiträge* zur Pandemiebekämpfung zu nutzen, um an die bisherigen gemeinsamen Leistungen anzuschließen und so sich selbst und andere zu schützen (*Identifikation*). Gesundheitsschutz und Infektionskontrolle werden dabei als – ausschließliche – *normative Ziele* der App betont.

### App-Nutzung als solidarische Bürger*innenpflicht

Auch im medialen Diskurs fungiert Solidarität als wesentlicher normativer Bezugspunkt. Als Beispiele sollen hier zwei Kommentare dienen, in denen die App-Nutzung als solidarische Bürger*innenpflicht gerahmt wird. So wirbt der Hauptstadt-Korrespondent der Deutschen Welle, Volker Witting, in einem Kommentar für die App-Nutzung und sieht darin die Möglichkeit, „[m]it Bluetooth ein Signal der Solidarität [zu] senden“ (Witting 2020). Er selbst habe die App aus Überzeugung installiert, denn „Menschenleben zu retten ist Bürgerpflicht“ (Witting 2020). Die App sei ein großer Schritt nach vorne im Kampf gegen die Pandemie (Witting 2020). Das Retten von Menschenleben und die Pandemiebekämpfung werden von ihm somit als normative Ziele der App ausgemacht, wobei das Verfolgen dieser Ziele als „Bürgerpflicht“ verstanden wird. Hier wird Solidarität also explizit in Zusammenhang mit dem Pflichtbegriff gebracht. Zugleich verweist Witting auf den Charakter der Freiwilligkeit, der die App durchziehe, wenn es etwa um das Anzeigen eigener Infektionen gehe (Witting 2020). Sowohl der Bezug auf die Bürger*innen wie auch die Betonung der Freiwilligkeit können hier als Identifikationsgrundlage gelten. Die App-Nutzung versteht er als wichtigen Beitrag der Pandemiebekämpfung. Schließlich hält Witting fest: „Die Corona-Warn-App ist also ein Stück gelebte deutsche Demokratie mit einem hohen Grad an Selbstverantwortung“ (Witting 2020). Dabei lässt sich der Bezug auf die „gelebte deutsche Demokratie“ als weiteres Identifikationspotenzial lesen, die Verbindlichkeit des solidarischen Beitrags wird im Verweis auf den „hohen Grad an Selbstverantwortung“ weiter ausgedeutet.

Auch ein Kommentar in der Schwäbischen Zeitung operiert mit ähnlichen Begriffen und offenbart ein Verständnis einer App-Nutzung als solidarischer Bürger*innenpflicht. Unter dem programmatischen Titel „Die freiwillige Nutzung der Corona-Warn-App ist eine Bürgerpflicht“ heißt es hier: *„Die Bewältigung der Corona-Krise war, ist und bleibt eine gesamtgesellschaftliche Herausforderung*. Da man dies also nur gemeinsam schafft, sollte jede(r) Einzelne es als Bürgerpflicht betrachten, jede sich bietende Möglichkeit im Kampf gegen das Virus zu nutzen – und somit auch die App. Eine Entscheidung eines jeden Einzelnen, zum Wohle und der Gesundheit aller“ (Wollny 2020; Hervorh. Orig.).

Hier tauchen paradigmatisch die unterschiedlichen Aspekte von Solidarität auf: Als *Solidaritätsgruppe* gilt die Gesamtgesellschaft, die hier mit der Gemeinschaft der Bürger*innen gleichgesetzt wird. Hier ist jede*r dazu aufgerufen, einen Beitrag zur Pandemiebekämpfung zu leisten, weil nur so die gemeinsame Aufgabe bewältigt werden kann. Die Interdependenz bzw. gemeinsame Betroffenheit aller Mitglieder der Gemeinschaft bildet somit den Ausgangspunkt und eine wesentliche *Identifikationsgrundlage*, um eine „Bürgerpflicht“ zum Leisten jedes möglichen *Beitrags* zu begründen, wozu auch die Nutzung der App gezählt wird. Als *normative Ziele* werden das Wohl und die Gesundheit aller ausgemacht.

### Zwischen der Warnung vor Entsolidarisierung und einem Plädoyer für eine umfassendere Solidarität

Während wir zum einen ein starkes Werben für die App, vereinzelt gar Forderungen einer Nutzungspflicht sehen (z. B. WELT 2020), finden sich andernorts Äußerungen, die offensiv die Freiwilligkeit der App anmahnen. Derartige Äußerungen greifen damit auch einen wichtigen Aspekt der fachwissenschaftlichen, ethischen Debatte auf und bewegen sich zwischen einer Warnung vor Entsolidarisierung und einem Plädoyer für umfassendere Solidarität.

Dies zeigt sich etwa auf der gemeinsamen Informationsseite der Verbraucherzentralen, die zwar vorrangig Informationen zu Funktionsweise und Datenschutz der App bereitstellt, aber dabei implizit auch ein bestimmtes Solidaritätsverständnis transportiert. So wird mehrfach angemahnt, dass – auch im Sinne der Akzeptanz – eine App-Pflicht auch nicht „durch die Hintertür“ eingeführt werden dürfe, da einige Verbraucher die App mangels Smartphones oder aktueller Betriebssysteme auf ihrem Smartphone nicht nutzen könnten (Verbraucherzentrale 2020). Man dürfe nicht „die Freiwilligkeit schleichend zum Zwang machen“, eine Nicht-Nutzung der App keine Nachteile für die Betroffenen bedeuten (Verbraucherzentrale 2020). Während bei anderen der Fokus darauf lag, möglichst viele Mitglieder der (Bürger*innen‑)Gemeinschaft als Nutzer*innen zu gewinnen, wird hier die Exklusivität dieser Gruppe thematisiert: Nur wer ein entsprechendes Endgerät besitzt, kann die App nutzen.

Dieser Aspekt der Freiwilligkeit und die Warnung vor einem „faktischen Zwang“ sind Gründe für regelmäßig geäußerte Forderungen nach einem Begleitgesetz zur Corona-Warn-App, die schließlich etwa im Vorschlag zu einem „Tracing-App-Freiwilligkeits- und Zweckbindungs-Gesetz“ der Bundestagsfraktion von BÜNDNIS 90/DIE GRÜNEN deutlich werden. Dieser richtet sich gegen eine Benachteiligung bei Nicht-Nutzung und sieht eine Sanktionierung bei entsprechenden Verstößen vor (Deutscher Bundestag 2020, S. 2). Im Entwurf wird Freiwilligkeit für eine nötige Akzeptanz der App vorausgesetzt, die nicht nur die Freiheit von rechtlichem, sondern auch von faktischem Zwang bedeute. Es dürfe keinen sozialen oder wirtschaftlichen Druck zur Nutzung sowie keine Nachteile oder finanziellen Einbußen bei Nicht-Nutzung geben (Deutscher Bundestag 2020, S. 1–2). Dieser starke Fokus auf die Freiwilligkeit rückt die Notwendigkeit einer eigenständigen Identifikation als wesentliche Grundlage für solidarisches Handeln in den Blick. Eine Verpflichtung der Bürger*innen, eine entsprechende App zu installieren und mit sich zu führen, sei auszuschließen (Deutscher Bundestag 2020, S. 7). Im Hintergrund steht hier die Befürchtung, dass z. B. Unternehmen die App für das Betreten von Geschäftsräumen verpflichtend machen und so faktisch eine Nutzung erzwingen (Deutscher Bundestag 2020, S. 7). Hingegen soll gesichert werden, dass die freiwillige App-Nutzung als „sinnvoller Baustein [unter anderen; NEG] zur Eindämmung und Bekämpfung der COVID-19-Pandemie“ (Deutscher Bundestag 2020, S. 1) beitragen kann. Dazu solle die Zweckbindung der App – die Kontaktnachverfolgung im Rahmen des Infektionsschutzes – explizit festgeschrieben werden (Deutscher Bundestag 2020, S. 1). Diese strikte Zweckbindung ist auch als Versuch zu lesen, das normativ wertvolle Ziel der App sicherzustellen.

Hier werden die einzelnen Elemente der Solidarität somit anders als zuvor gewichtet: Zwar wird der *Gruppenbezug* nicht weiter ausbuchstabiert, der Verweis auf das Risiko einer Benachteiligung von Nicht-Nutzer*innen macht jedoch die potenzielle Exklusivität einer Solidaritätsgruppe zum Thema. Diese betrifft diejenigen, die die App nicht nutzen wollen, und die, die sie mangels entsprechender Ressourcen nicht nutzen können. Die App-Nutzung als einer von vielen solidarischen *Beiträgen* soll freiwillig und nicht verpflichtend sein, die eigenständige *Identifikation* mit dem Ziel der App wird in diesem Sinne stark gemacht. Eine erzwungene Nutzung würde hingegen dieser Identifikation und damit einer wesentlichen Grundlage für Solidarität entbehren. Ebenso wird das *normative Ziel* der App durch die Zweckbindung in den Mittelpunkt gerückt. Die Warnung vor Nachteilen bei Nicht-Nutzung und der starke Fokus auf das normativ wertvolle Ziel sind dabei einerseits als eine Warnung vor Entsolidarisierung, andererseits als Plädoyer für eine normativ gehaltvolle Solidarität zu lesen, die möglichst viele einschließt.

### Der besondere Schutz der vulnerablen Gruppen als „ein Gebot der Solidarität“

Lag in den bisherigen Beispielen der Fokus auf Solidarität *innerhalb* einer bestimmten Gruppe, finden sich in der Debatte auch Appelle an die Solidarität *mit* einer bestimmten Gruppe. Diese klingen etwa in den Äußerungen des Diakonie-Präsidenten Ulrich Lilie im „Diakonie-Zitat“ und in einem umfassenden Blogbeitrag an. Diese Äußerungen des Präsidenten eines der sechs Spitzenverbände der freien Wohlfahrtspflege stellen zudem ein wichtiges Beispiel für die Verwendung des Solidaritätsbegriffs im zivilgesellschaftlichen Diskurs dar.

So steigt das „Diakonie-Zitat“ unmittelbar mit der Feststellung ein, dass die Nutzung der Corona-Warn-App ein „Gebot der Solidarität“ sei (Lilie 2020a). Sie könne dazu beitragen „in größtmöglicher Normalität mit dem Virus zu leben“ (Lilie 2020a). Gemeinsam mit weiteren Maßnahmen könnten so eine neue Infektionswelle und ein neuer Lockdown mit erheblichen Freiheitseinschränkungen vermieden werden (Lilie 2020a). Die App-Nutzung wird somit als wichtiger Beitrag in der Pandemiebekämpfung eingeordnet, der solidarisch geboten ist. Als normatives Ziel wird von Lilie dabei die Infektionskontrolle sowie ein möglichst „normales“ Leben mit wenig Freiheitseinschränkungen benannt. Voraussetzung dafür sei, dass „genug Menschen mitmachen“ (Lilie 2020a). Lilie hält fest, dass diese Ziele im Interesse aller seien, jedoch „in ganz besonderer Weise im Interesse hoch vulnerabler Gruppen“ (Lilie 2020a). Diese seien – so heißt es auf seinem Blog – „weiterhin darauf angewiesen, sich zu schützen“ (Lilie 2020b). Hier werden zum einen zwei wesentliche Identifikationsmöglichkeiten für das solidarische Handeln aufgerufen, indem *erstens* das eigene Interesse an einer App-Nutzung deutlich und *zweitens* für die Situation der Vulnerablen sensibel gemacht wird. Zum anderen fällt der eher transitive Solidaritätsbezug auf, indem die hoch vulnerablen Gruppen als spezielle Adressatinnen der Solidarität herausgestellt werden. Im Anschluss mahnt Lilie die Barrierefreiheit der App an: Es sei wichtig, diese auch Menschen mit Behinderungen, im höheren Lebensalter und ohne deutsche Sprachkenntnisse zugänglich zu machen (Lilie 2020a). Lilie weist damit auf Barrieren zur Teilnahme am solidarischen Handeln und auf entsprechend notwendige Ressourcen, Positionen oder Kompetenzen hin.

Ein solches Solidaritätsverständnis changiert in seinem *Gruppenbezug *zwischen einen eher intransitiven Rekurs – gemeinsam den Lockdown für alle verhindern – und einem transitiven Gebrauch des Begriffs, wonach speziell vulnerable Gruppen zu schützen sind. Die App wird in beiden Fällen als wichtiger *Beitrag* unter anderen gesehen, um Gesundheit und Freiheit zu bewahren sowie möglichst normal mit dem Virus zu leben. Es fällt auf, dass ergänzend zu diesen *normativen Zielen*, in der Rede vom Gebot der Solidarität ein Verständnis eines nicht weiter begründungsbedürftigen, wie auch immer gearteten intrinsischen Werts der Solidarität anklingt. Als *Identifikationsgrundlage* wird v. a. das eigene Interesse der individuellen Nutzer*innen benannt. Zugleich appelliert Lilie an die Empathie und die sorgende Haltung gegenüber den besonders Vulnerablen und ruft so weitere Identifikationsmöglichkeiten für Solidarität mit anderen auf.

## Diskussion und Ausblick: Mit Bluetooth ein Signal *welcher* Solidarität senden?

In der öffentlichen Debatte werden die einzelnen Elemente der Solidarität sehr unterschiedlich gefüllt und ins Verhältnis zueinander gesetzt. So unterscheiden sich die Verwendungsweisen nicht nur hinsichtlich der *Identifikationsgrundlage* (Zugehörigkeit zu einer Gemeinschaft, Eigeninteresse, Empathie für andere etc.) und der Inklusivität der *Gruppe* (abgeschlossene Bürger*innengemeinschaft, Teilhabebarrieren usw.). Auch mit Blick auf die Verbindlichkeit des *Beitrags* zeigt sich diese Heterogenität. Während die einen Solidarität in Zusammenhang mit dem Pflichtbegriff bringen oder von einem Gebot sprechen, wird bei anderen die Unverbindlichkeit betont. Ebenso werden unterschiedliche *normative Ziele* angeführt, die im Zuge des solidarischen Handelns erreicht werden sollen. Angesichts der Allgegenwart und Vielfalt der Solidaritätsrekurse mit unterschiedlichen praktischen Konsequenzen wird nachvollziehbar, warum Netzaktivist*innen in der Debatte um die Corona-Warn-App eine „unwillkürliche Instrumentalisierung von Solidarität“ (digitalcourage.de 2020) befürchten.

Vor diesem Hintergrund zeigt sich die Notwendigkeit, mit weiterführenden Maßstäben die einzelnen Solidaritätsrekurse zu analysieren und zu beurteilen, inwiefern eine bestimmte Verwendungsweise moralphilosophisch gedeckt ist und wann eine missbräuchliche, „parasitäre“ (Scholz 2008) Verwendung des Solidaritätsbegriffs vorliegt. Dabei gilt es v. a. zu fragen, wann die einzelnen Elemente von Solidarität in ein solches Missverhältnis geraten, dass – trotz ihres formalen Vorliegens – kaum mehr von moralisch gehaltvoller Solidarität gesprochen werden kann. Als solche weiterführenden Maßstäbe lassen sich zum Beispiel die vier normativen Kriterien einer kontextsensitiven, moralisch gehaltvollen Solidaritätskonzeption verstehen, die ich an anderer Stelle formuliert habe (vgl. dazu Ellerich-Groppe 2021).

Ein *erstes* Kriterium weist auf die Notwendigkeit einer *Solidaritätsoffenheit* hin, die auf individueller und struktureller Ebene die für Solidarität notwendige Identifikation anregen und ermöglichen soll. Ein *zweites* Kriterium zollt der für Solidarität notwendigen Exklusivität und Abgrenzung Tribut, mahnt jedoch zugleich die *Gestaltbarkeit der Inklusivität *der Solidaritätsgruppe an. Die *Angemessenheit des Beitrags* als *drittes* Kriterium nimmt für solidarische Beiträge eine gewisse Verbindlichkeit an, weist jedoch die Etablierung unausgewogener, einseitiger Herrschaftsverhältnisse zurück. *Viertens* betont die *normative Abhängigkeit* die Notwendigkeit einer Bezugnahme auf ein moralisch wertvolles Ziel (und eben nicht nur politische oder ökonomische Interessen). Mithilfe dieser Kriterien lassen sich für alle vorgestellten Solidaritätsrekurse kritische Rückfragen formulieren. Die wichtigsten sollen nun kurz skizziert werden.

So wirft ein Verständnis der Pandemiebekämpfung als Gemeinschaftsprojekt der Bürger*innen zunächst die Frage auf, wer eigentlich zu den Mitbürger*innen – und damit zur Solidaritätsgruppe – zählt. Dass nicht jede*r Mitbürger*in ein zur App-Nutzung fähiges digitales Endgerät besitzt, stellt eine weitere Exklusionsgefahr dar, die es zu vermeiden gilt. Mit Blick auf die Angemessenheit bzw. Verbindlichkeit des solidarischen Beitrags ist v. a. eine Stelle in Merkels Videoansprache interessant. Wenn Merkel an die Mitbürger*innen appelliert und die Nutzung der Corona-Warn-App als Fortsetzung eines bereits erfolgten solidarischen Handelns rahmt, kann dies zumindest für diejenigen, die sich als Teil der Bürger*innengemeinschaft identifizieren, trotz aller Hinweise auf die Freiwilligkeit der App eine verbindliche Wirkung entfalten. Das Kriterium der normativen Abhängigkeit mag erklären, warum Merkel in ihrer Videobotschaft daran gelegen ist, durch den Verweis auf IT-Sicherheit, Transparenz und Datenschutz keinen Zweifel an dem ausschließlich normativ wertvollen Ziel der App aufkommen zu lassen – etwas, das gerade für politische Entscheidungsträger*innen von entscheidender Bedeutung ist und die Relevanz der Frage unterstreicht, *wer* eigentlich warum zu Solidarität aufruft.

Die Verbindlichkeit des solidarischen Beitrags wird im Zuge des Framings der App-Nutzung als Bürger*innenpflicht zwar direkt mit dem Pflichtbegriff in Zusammenhang gebracht, zugleich wird aber die absolute Freiwilligkeit der App und der hohe Grad an Selbstverantwortung betont. Wie verbindlich die App-Nutzung ist, bleibt dadurch schlussendlich unklar. Das Retten von Menschenleben als normatives Ziel stellt ohne Frage ein moralisch wertvolles Anliegen dar. Allerdings handelt es sich hier weniger um eine solidarische denn um eine weiterreichende moralische Verantwortlichkeit, die nicht allein von Solidarität abhängen sollte. Die Interdependenz und gemeinsame Betroffenheit vom Virus können grundsätzlich eine identifikationsstiftende Gemeinsamkeit sein, drohen aber in ihrer Allgemeinheit nur wenig konkrete Identifikation anzuregen.

Der *Entwurf für ein Corona-Warn-App-Begleitgesetz *nimmt seinen Ausgangspunkt bei der Exklusivität der Solidaritätsgruppe. Er mahnt insofern eine inklusive Gestaltung der App an, als er auf die Gefahren von Diskriminierung und Benachteiligung hinweist, wenn Menschen von der Nutzung ausgeschlossen sind und etwa Unternehmen das Betreten ihrer Geschäftsräume an eine installierte App knüpfen. Im Sinne der Solidaritätsoffenheit lassen sich ambivalente Konsequenzen feststellen: Während der Gesetzentwurf einerseits darauf angelegt ist, auf struktureller Ebene eine freiwillige Identifikation als wesentlichen Ausgangspunkt von Solidarität zu befördern und vor Zwang zu schützen, ist er mangels konkreter Identifikationspunkte andererseits auf individueller Ebene wenig identifikationsfördernd. In der Folge muten die Verbindlichkeit und das *commitment* zum Leisten des solidarischen Beitrags eher schwach an. Mit seinem starken Fokus auf die Zweckbindung macht dieser Solidaritätsrekurs jedoch das Element der normativen Abhängigkeit besonders überzeugend stark.

Das transitivere Verständnis von *Solidarität zum besonderen Schutz der Vulnerablen *bietet gleich mehrere nachvollziehbare Identifikationspotenziale und kann in diesem Sinne helfen, Solidaritätsoffenheit auf individueller Ebene zu befördern. Zugleich ruft Lilie in seinem Beitrag – wohlgemerkt mit guten Absichten im Sinne der Barrierefreiheit – gesellschaftlich verankerte Altersbilder und Stereotype auf, die eine Identifikation zunächst erschweren oder verhindern können. Mit Blick auf die normative Abhängigkeit lassen sich zwei Rückfragen stellen: Zum einen ist es problematisch, wenn in der Rede von der App-Nutzung als „Gebot der Solidarität“ zunächst ein quasi intrinsischer Wert von Solidarität aufgerufen wird. Zum anderen macht der Rekurs auf eine Vielzahl normativer Prinzipien im weiteren Verlauf der Äußerung eine Abwägung notwendig, welches Prinzip im Zweifel ausschlaggebend ist. Gerade wenn dabei Konflikte auftreten, bleibt die Frage nach dem *entscheidenden *Ziel offen.

So einig sich somit die Autor*innen darin scheinen, durch die App „[m]it Bluetooth ein Signal der Solidarität senden“ (Witting 2020) zu können, so unterschiedlich sind ihre Vorstellungen, wer genau hier an wen sendet und wie stark dieses Signal ist. Das bestätigt den Eindruck, dass das Solidaritätskonzept in der vorliegenden Debatte sehr unterschiedlich modelliert, modifiziert und modularisiert wird. Zudem scheinen einzelne Aspekte der verschiedenen Solidaritätsrekurse aus der Perspektive eines moralisch gehaltvollen Solidaritätsverständnisses problematisch.

Der differenzierte Blick auf die einzelnen Verwendungsweisen von Solidarität in der öffentlichen Debatte zur Corona-Warn-App ist ein Gewinn der vorliegenden Analyse, die insgesamt in mindestens dreierlei Hinsicht einen Mehrwert bieten kann:

*Erstens* werden konkrete Anknüpfungspunkte deutlich, um den solidarischen Charakter der Corona-Warn-App selbst abzuschätzen. Hier gilt: Je umfassender die normativen Kriterien einer moralisch gehaltvollen Solidaritätskonzeption erfüllt sind, desto solidarischer ist eine solche App. In diesem Zuge können punktuell konkrete Potenziale für eine solidarischere Gestaltung der App aufgezeigt werden. So könnten zielgruppenspezifische Werbemaßnahmen und Schulungen Solidaritätsoffenheit fördern, niedrigschwellige App-Nutzungsmöglichkeiten etwa durch kostenlose Bluetooth-Armbänder oder kostenlose, für die App-Nutzung optimierte Smartphones (Loi 2021) die Inklusivität steigern. Die Ergebnisse der Analyse der Debatte können so Anregungen und Ergänzungen für bereits vorliegende ethische Analysen, Einordnungen und Richtlinien von Corona-Warn-Apps sein (vgl. etwa Lucivero et al. 2020; Morley et al. 2020; Ranisch et al. 2021).

*Zweitens *kann die Analyse die Verwendungsweisen des Solidaritätsbegriffs selbst einer differenzierten Kritik unterziehen. So zeigt sich, dass sich die Solidaritätsrekurse – je nachdem welches Element von Solidarität wie gewichtet wird – erheblich unterscheiden und sehr heterogene Implikationen haben können. In diesem Zuge kommt auch der Frage, wer aus welchen Gründen zu Solidarität aufruft oder an diese appelliert, eine wesentliche Bedeutung zu. Die normativen Kriterien einer kontextsensitiven, moralisch gehaltvollen Solidaritätskonzeption stellen eine erste Grundlage dar, um die jeweiligen Voraussetzungen und normativen Implikationen der Solidaritätsrekurse explizit zu machen sowie gerechtfertigte von parasitären Verwendungsweisen von Solidarität zu unterscheiden. Dies erscheint in der Corona-Pandemie, in der die Rede von Solidarität allgegenwärtig ist, besonders relevant. Angesichts des theoriegeleiteten Samples der vorliegenden Analyse sollte dieses Anliegen auch im Rahmen einer umfassenderen Diskursanalyse zur Corona-Warn-App weiterverfolgt werden. Ebenso scheinen derartige Analysen auch für weitere Themenschwerpunkte wie die Generationenbeziehungen vielversprechend (vgl. Ellerich-Groppe et al. 2021). Gleiches gilt für weitere medizinethische Debatten mit Solidaritätsbezug, etwa zu Self-Tracking im Gesundheitswesen, öffentlicher Gesundheitsversorgung und Organspende. Auch hier können die Kriterien ihr Klärungspotenzial entfalten und dazu beitragen, dass sich die Macht der Solidarität tatsächlich entfalten kann.

Schließlich werden *drittens* die Grenzen von Solidaritätsrekursen ersichtlich. Die eigenständige Identifikation erweist sich mit Blick auf die Corona-Warn-App als Kernelement von Solidarität, das im regelmäßigen Verweis auf die Freiwilligkeit der App hervorgehoben wird. Solidarität kann nicht erzwungen oder angeordnet werden, vielmehr kann um sie lediglich geworben oder daran appelliert werden – und so vielleicht die notwendige Offenheit geschaffen werden. Eine weitere Limitation liegt in der Frage, welche Beiträge und Verantwortlichkeiten sinnvollerweise solidarisch genannt werden können. Nicht jeder sinnvolle Beitrag zur Pandemiebekämpfung ist zwangsläufig solidarisch, manche Verantwortlichkeiten – etwa das Retten von Menschenleben – ergeben sich schon aufgrund weiterreichender moralischer Verantwortlichkeiten. Schließlich macht die Debatte deutlich, dass nicht jede Person jeden Beitrag ohne weiteres leisten kann. Diese Begrenztheit der für einen solidarischen Beitrag notwendigen Kompetenzen, Ressourcen und Kapazitäten gilt es gerade in einer lang andauernden Krise wie der Corona-Pandemie mehr zu berücksichtigen.
